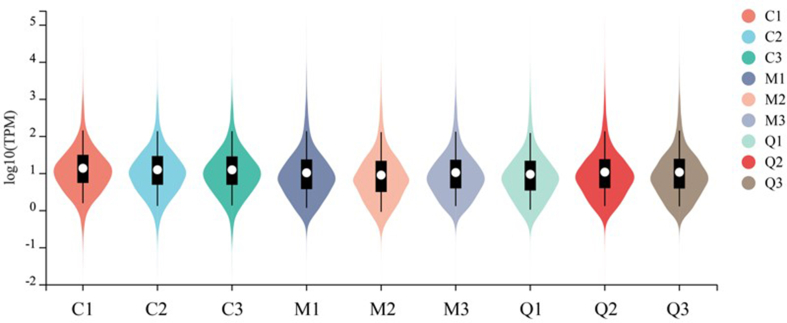# Suppressing inflammatory signals and apoptosis-linked sphingolipid metabolism underlies therapeutic potential of Qing-Jin-Hua-Tan decoction against chronic obstructive pulmonary disease

**DOI:** 10.1016/j.heliyon.2024.e24336

**Published:** 2024-01-18

**Authors:** Jing Yang, Xin Shen, Mi Qin, Ping Zhou, Fei-Hong Huang, Yun You, Long Wang, Jian-Ming Wu

**Affiliations:** a Department of Pharmacy, Chengdu Fifth People's Hospital, Chengdu University of Traditional Chinese Medicine, Chengdu 611130, PR China; b Department of Traditional Chinese Pharmacy, Chengdu First People's Hospital, Chengdu 610041, PR China; c School of Pharmacy, Southwest Medical University, Luzhou 646000, PR China; d Institute of Chinese Materia Medica, China Academy of Chinese Medical Sciences, Beijing 100700, PR China; e School of Basic Medical Sciences, Southwest Medical University, Luzhou 646000, PR China

**Keywords:** Qing-Jin-Hua-Tan decoction, Omics, Apoptosis, Inflammation, Chronic obstructive pulmonary disease

## Abstract

**Background:**

Qing-Jin-Hua-Tan decoction (QJHTD) is a classic traditional Chinese medicine (TCM) prescription that first appeared in the ancient book Yi-Xue-Tong-Zhi. QJHTD has shown effectiveness for treating chronic obstructive pulmonary disease (COPD), although its mechanisms of action are still perplexing. The molecular mechanisms underlying the curative effects of QJHTD on COPD is worth exploring.

**Methods:**

*In vitro* antiapoptotic and antiinflammatory activities of QJHTD were evaluated using cell viability, proliferation, apoptosis rate, and expression of IL-1β and TNF-α in BEAS-2B and RAW264.7 cells challenged with cigarette smoke (CS) extract (CSE) and lipopolysaccharide (LPS). *In vivo* therapeutic activities of QJHTD were evaluated using respiratory parameters (peak inspiratory flow (PIFb) and peak expiratory flow (PEFb) values), histopathology (mean linear intercept, MLI), and proinflammatory cytokine (IL-1β and TNF-α) and cleaved caspase-3 (c-Casp3) levels in the lung tissue of CS–LPS-exposed BALB/c mice. Network pharmacology-based prediction, transcriptomic analysis, and metabolic profiling were employed to investigate the signaling molecules and metabolites pertinent to the *anti*-COPD action of QJHTD.

**Results:**

Increased cell viability and proliferation with decreased apoptosis rate and proinflammatory cytokine expression were noted after QJHTD intervention. QJHTD administration elevated PEFb and PIFb values, reduced MLI, and inhibited IL-1β, TNF-α, and c-Casp3 expression *in vivo*. Integrated network pharmacology–transcriptomics revealed that suppressing inflammatory signals (IL-1β, IL-6, TNF, IκB–NF-κB, TLR, and MAPK) and apoptosis contributed to the *anti*-COPD property of QJHTD. Metabolomic profiling unveiled prominent roles for the suppression of apoptosis and sphingolipid (SL) metabolism and the promotion of choline (Ch) metabolism in the *anti*-COPD effect of QJHTD. Integrative transcriptomics–metabolomics unraveled the correlation between SL metabolism and apoptosis. *In silico* molecular docking revealed that acacetin, as an active compound in QJHTD, could bind with high affinity to MEK1, MEK2, ERK1, ERK2, Bcl2, NF-κB, and alCDase target proteins.

**Conclusion:**

The therapeutic effect of QJHTD on COPD is dependent on regulating inflammatory signals and apoptosis-directed SL metabolism. These findings provide deeper insights into the molecular mechanism of action of QJHTD against COPD and justify its theoretical promise in novel pharmacotherapy for this multifactorial disease.

## 1 Introduction

Increasing attention is being paid to chronic obstructive pulmonary disease (COPD)—a weakening condition that has become the third leading cause of morbidity and mortality for both genders worldwide [1]. COPD is a common respiratory disease distinguished by airway limitation and airflow obstruction. The lesions in this disease manifest as persistent small airway inflammation and progress with incomplete reversibility [2]. Clinically, a series of typical signs, including chronic cough, sputum production, wheezing, dyspnea, and even respiratory failure, represent the outcomes of COPD progression with worsening of lung function [3]. When such alterations occur as worsening dyspnea and increased sputum volume (specifically purulent sputum), acute exacerbation of COPD can be diagnosed in the clinical setting [4]. Furthermore, these symptoms are linked to the pathological severity of COPD; airway inflammation (infective or non-infective) followed by an imbalance between lung injury and repair determines disease prognosis. Therefore, attenuating airway inflammation and improving lung recovery are expected to play indispensable roles in COPD treatment. The recommended pharmacological therapies for COPD involve the application of bronchodilators, antiinflammatory agents, antiinfective agents, and combination agents [5]. These medications can produce significant effects on alleviating respiratory symptoms, particularly in the short-term treatment of COPD. However, several adverse reactions, such as tachycardia, tremor, vomiting, diarrhea, hyperglycemia, hypertension, fluid retention, and osteoporosis, restrict extensive applications of those pharmacological modalities to COPD [6]. Moreover, drug resistance due to prolonged medications in patients may result in disease relapse [7]. Thus, the development of additional drug regimens that are more effective and that have fewer side effects is urgently needed.

Traditional Chinese medicine (TCM) formulas offer a promising alternative to routine therapeutic drugs. Qing-Jin-Hua-Tan decoction (QJHTD), a classical Chinese herbal prescription recorded in the ancient (Ming dynasty's) Chinese medical book Yi-Xue-Tong-Zhi, has been used to treat various lung disorders, including COPD, for almost 500 years. This formula comprises 11 medicinal herbs as listed in Table 1. In TCM theory, the formula's traditional efficacy is described as clearing lung heat and eliminating phlegm. These conventional terms can be translated into the pharmacological activities of suppressing inflammation, relaxing bronchial smooth muscle, facilitating expectoration, alleviating coughs, and regulating immunity. In the clinic, QJHTD and its modified prescriptions have been administered to treat inflammatory bronchopneumopathies as diverse as acute/chronic bronchitis, bronchiectasis, pneumonia, and COPD, which pertain to the TCM syndrome of phlegm heat accumulation in the lung. This formula holds tremendous potential for the treatment of COPD associated with prolonged airway inflammation. Nevertheless, the molecular mechanism of action of QJHTD in COPD remission remains elusive.

**Table 1:** Composition of QJHTD.

Medicinal part/name	Chinese name	Origin (Latin scientific name)	Portion dosage (g)
*Radix scutellariae* (*R. Scutellariae*)	Huangqin	*Scutellaria baicalensis* Georgi	5.595
*Fructus gardeniae* (*F. Gardeniae*)	Zhizi	*Gardenia jasminoides* J.Ellis	5.595
*Rhizoma anemarrhenae* (*R. Anemarrhenae*)	Zhimu	*Anemarrhena asphodeloides* Bunge	3.73
*Cortex mori* (*C. Mori*)	Sangbaipi	*Morus alba* L.	3.73
*Semen trichosanthis* (*S. Trichosanthis*)	Gualouren	*Trichosanthes kirilowii* Maxim. or *Trichosanthes rosthornii* Harms	3.73
*Bulbus fritillariae thunbergii* (*B.F. Thunbergii*)	Zhebeimu	*Fritillaria thunbergii* Miq.	3.73
*Radix ophiopogonis* (*R. Ophiopogonis*)	Maidong	*Ophiopogon japonicus* (Thunb.) Ker Gawl.	3.73
*Exocarpium citri rubrum* (*E.C. Rubrum*)	Juhong	*Citrus reticulata* Blanco	3.73
*Sclerotium poriae cocos* (*S.P. Cocos*)	Fuling	*Poria cocos* (Schw.) Wolf	3.73
*Radix platycodonis* (*R. Platycodonis*)	Jiegeng	*Platycodon grandiflorus* (Jacq.) A.DC.	7.46
*Radix glycyrrhizae* (*R. Glycyrrhizae*)	Gancao	*Glycyrrhiza uralensis* Fisch.	1.492

In this study, the *anti*-COPD activities of QJHTD *in vitro* and *in vivo* were validated. The molecular mechanisms of QJHTD therapy using network pharmacology were further decrypted. Transcriptomics and metabolomics in the *in vivo* exploration identified differentially activated pathways and distinctively abundant metabolites (endogenous and exogenous), respectively. Further relational analysis revealed drug-triggered and gene-governed metabolic pathways. Finally, virtual molecular docking hinted at the potential active components in the preparation of QJHTD. The findings serve to decode the medicinal properties of QJHTD, underpinning the clinical application of traditional medication to complex respiratory disorders.

## 2 Materials and methods

### 2.1 Drug preparation and constituent analysis

QJHTD samples (lyophilized powder, Lot No. Z200601) were kindly provided by Ji-Ren Pharmaceutical Group Co., Ltd (Bozhou, China). This recipe contains 11 herbal materials and their quantities, listed in Table 1, which were chosen according to the conversion of ancient doses into modern routine administration. The whole prescription thus amounted to 46.252 g in mass of the prepared slices. The final yield of the dried extract was 29.2 %. Main components of QJHTD were analyzed with baicalin (150 μg mL^−1^) as the reference standard and its chemical features were fingerprinted using high-performance liquid chromatography (HPLC). The operating conditions were shown in Table 2. The gradient elution is programmed in Table 3.

**Table 2:** Chromatographic conditions in analysis of QJHTD components.

Parameter	Value
Sample injection volume	5 μL
Separating column	Agilent Eclipse XDB C_18_ column (250 mm × 4.6 mm, 5 μm)
Column temperature	30 °C
Mobile phase A	acetonitrile
Mobile phase B	0.1 % phosphoric acid solution
Flow rate	1 mL min^−1^
Detection wavelength	230 nm
Theoretical plate number	≥2500 (as calculated upon baicalin peak)

**Table 3:** Gradient elution for constituent detection of QJHTD extract.

Elution time (min)	A: acetonitrile (%)	B: 0.1 % phosphoric acid solution (%)
0	5	95
5	6	94
25	15	85
35	21	79
37	24	76
48	25	75
53	26	74
56	28	72
65	100	0
76	100	0

Dexamethasone (DEX; Lot No. C84-1434-1G, purity >99 %) as a positive control was purchased from Dalian Meilun Biotech Co., Ltd (Dalian, China).

All agents were thoroughly dispersed in culture medium or deionized water at specific concentrations when used for *in vitro* or *in vivo* activity testing.

### 2.2 Preparation and quality control of cigarette smoke extract (CSE)

BEAS-2B and RAW264.7 cells were stimulated by CSE and lipopolysaccharide (LPS) to evaluate the *in vitro* therapeutic effect of QJHTD on COPD [8]. For CSE preparation, a commercial cigarette (Wuniu, China Tobacco Sichuan Industrial Co. LTD, Sichuan, China) containing 10 mg tobacco tar, 0.7 mg nicotine, and 11 mg carbon monoxide was burnt to produce cigarette smoke (CS). The resultant smoke was then bubbled through 20 mL of serum-free high-glucose DMEM (hgDMEM) (Gibco, NY, USA) in a 50 mL conical tube under continuous negative pressure using a vacuum pump. After cigarette burning-off and complete dissolution of the smoke, the smoke solution was blended upside down and subsequently passed through a 0.22 μm membrane filter (Millipore, Billerica, MA, USA). This filtrate represented 100 % (relative concentration) CSE, which was diluted with serum-free hgDMEM to the required concentration. Fresh CSE was prepared for each experiment.

Following the same preparation procedure, the absorbance of CSE at 320 nm (OD_320_) was measured at five different time points to determine its stability. The OD_320_ values of freshly prepared CSEs were 0.60, 0.62, 0.58, 0.59, and 0.59, yielding a mean ± SD of 0.60 ± 0.01. The coefficient of variation expressed as $\frac{\text{SD}}{\text{mean}}\times 100\%$ was 1.67 % (i.e., less than 5 %). Thus, stable CSE was obtained through the preparation method and was suitable for *in vitro* modeling.

### 2.3 Cell culture and In vitro COPD modeling

BEAS-2B and RAW264.7 cells were purchased from the American Type Culture Collection [9] and used for *in vitro* modeling of COPD. The two cell lines were cultured under uniform conditions as reported previously [10]. During the logarithmic growth phase, BEAS-2B and RAW264.7 cells were seeded in 96-well and 6-well plates at densities of 6 × 10^3^ and 2 × 10^5^ cells per well, respectively. After cell adherence, CSE was combined with LPS for stimulation. At 24, 48, and 72 h post-irritant challenge, BEAS-2B cell viability was measured with the MTT assay (Beyotime, Sichuan, China) to evaluate mimetic tracheobronchial epithelial injury. At 6, 12, 24, 48, and 72 h after CSE–LPS costimulation, extracellular inflammatory cytokines from RAW264.7 cells were determined using the ELISA kits (4A Biotech, Beijing, China) to assess mimetic airway inflammation. Together, the exploration of *in vitro* COPD modeling uncovered the optimal stimulating conditions.

### 2.4 Cell viability assessment

BEAS-2B cells in the logarithmic phase were seeded in a 96-well plate and divided into control, model (irritant challenge), DEX (40 μM), and QJHTD (12.5, 25, and 50 μg mL^−1^) groups. The cells were pretreated with the corresponding drugs or equivoluminal medium after cell attachment. Following pretreatment for 1 h, the cells, except for controls, were exposed to the irritant containing 5 % CSE and 1 μg mL^−1^ LPS with the uninterrupted medication. After 24 h of irritant challenge with concurrent treatment, the viability of BEAS-2B cells was examined to evaluate the cell-protective activity of QJHTD. Three replicates were used per test, and the experiment was repeated three times.

### 2.5 EdU cell proliferation assay

According to the manufacturer's instructions for the EdU Imaging Kits (Cy3) (APExBIO, TX, USA), BEAS-2B cells at a density of 6 × 10^3^ cells per well were incubated with EdU solution in 96-well plates after 24 h of CSE–LPS coexposure along with medicinal intervention [11]. Following cell fixation, membrane permeabilization, and click reaction in turn, DNA synthesis in the cells was examined using the ImageXpress Micro 4 Widefield High-Content Analysis System (Molecular Devices, Sunnyvale, CA, USA) [12] to assess cell proliferation activity. The experiment was repeated three times.

### 2.6 Cell apoptosis detection using TUNEL staining

In the present study, the TUNEL kit (TransGen Biotech, Beijing, China) was employed to observe apoptotic morphology. The procedure was as reported previously [10]. Fluorescence intensity was measured under a fluorescence microscope (Nikon Eclipse Ts2R, NY, USA). The experiment was repeated three times.

### 2.7 Fluorescence-activated cell Sorting (FACS) to confirm apoptosis

The FACS assay gave access to the quantitative analysis of apoptotic cells. Briefly, after grouping, modeling, and intervention as described above, BEAS-2B cells were harvested into 1.5 mL Eppendorf tubes and rinsed with precooled PBS three times. The cells in each tube were resuspended in 100 μL of 1 × binding buffer by gentle mixing. Thereafter, 3 μL of Annexin V-FITC and 3 μL of PI were added, and the cells were incubated for 10 min. The cell suspensions were transferred to tubes containing 200 μL of 1 × binding buffer, with gentle blending to test apoptotic cell percentage using a flow cytometer (BD Biosciences, San Jose, CA, USA). The experiment was repeated three times.

### 2.8 Proinflammatory cytokine measurement using ELISA

Since lung macrophages are implicated in bronchoalveolar inflammation, particularly during acute exacerbation of COPD, we considered RAW264.7 cells acceptable to model the key link in inflammatory damage to small airways. Specifically, RAW264.7 cells were seeded into 6-well plates at a density of 2 × 10^5^ cells per well. Upon adherence, the cells in each well were treated with the same drugs or medium. After 1 h, the cells separately underwent the same CSE–LPS costimulation and corresponding medication as mentioned above. ELISA kits (4A Biotech, Beijing, China) were used to determine the expression of the proinflammatory cytokines IL-1β and TNF-α in the culture supernatant as per the manual [10]. Absorbance post reaction at 450 nm (OD_450_) was measured using a microplate reader (BioTek Cytation 3, MA, USA) and transformed to protein content on the basis of the OD_450_-*versus*-concentration standard curve. The experiment was repeated three times.

### 2.9 Animal housing and In vivo COPD modeling

Animal experiments in the present study were authorized by the Center for Experimental Animal of Southwest Medical University, and all experimental procedures were approved by the Ethics Committee of Southwest Medical University [13] (license No. 20180309091). Male BALB/c mice, aged 6–8 weeks and weighing 18–22 g, were purchased from SpePharm (Beijing) Lab Animals Technology Co., Ltd (license No. SCXK (Beijing) 2019-0010; China). The animals were housed under well-controlled ambient conditions [14]. Sterile water and standard chow were provided *ad libitum*.

After 7 days of acclimation, the mice were randomly split into 6 groups (8 mice per group): control, model, DEX (1 mg kg^−1^), low-dose QJHTD (1.12 g kg^−1^), moderate-dose QJHTD (2.24 g kg^−1^), and high-dose QJHTD (4.48 g kg^−1^). Before irritant challenge, pulmonary function in all mice was detected at baseline. For COPD modeling, all the animals except controls were submitted to LPS and CS irritation as described previously [15]. The animal experiment lasted 42 days (Fig. 3A). On days 1 and 14, the mice for modeling were anesthetized with sodium pentobarbital (50 mg kg^−1^; Sigma‒Aldrich, MO, USA) and intratracheally injected with LPS (20 μg per mouse). The rest of the experimental duration was scheduled for mouse exposure to CS in a self-made instrument. The daily CS stimulus was controlled three times by burning six cigarettes, each for 30 min. One hour prior to the daily modeling, the mice in each group were intragastrically administered the corresponding drug or equivoluminal vehicle. During the *in vivo* modeling with drug administration, the respiratory status, fur glossiness, body mass, and survival rate of the mice were daily observed and recorded. Pulmonary function parameters such as peak inspiratory flow (PIFb) and peak expiratory flow (PEFb) of the mice were detected every 7 days using the FinePointe Whole Body Plethysmograph (WBP) system (DSI, MN, USA).

After the final assessment of lung function, all mice were euthanized to collect lung tissue and blood samples, which were stored in an ultralow temperature freezer (Thermo Scientific, MA, USA) or fixed with 4 % paraformaldehyde solution (Biosharp, Hefei, China) for subsequent analysis.

### 2.10 Pulmonary function test

In the *in vivo* study, the FinePointe WBP system was employed to test pulmonary function in mice. On the basis of the operating instructions, two time parameters, “Duration of Acclimation Period” and “Response Time”, were set to 5 and 3 min, respectively. Then, the mice were individually placed in the test chambers. The values of PIFb and PEFb were measured to represent ventilatory capacity.

### 2.11 Lung histopathology

The lung tissue from the mice was collected and fixed in 4 % paraformaldehyde solution for 24 h [16]. To analyze lung histopathology, we prepared paraffin-embedded sections of the lung tissue, deparaffinized the sections, and stained the tissue with hematoxylin plus 1 % eosin ethanol (H&E) solution. The stained histopathological sections were observed under a microscope (Nikon Eclipse TS100, Tokyo, Japan) and photographed.

The mean linear intercept (MLI), a measure of airspace enlargement (emphysema), was quantified to interpret lung pathology in the COPD mice. As reported by a previous study [17], 20 equidistant parallel pipelines were depicted on H&E-stained section images. Their intersection points with air-cavity walls (represented as Ns) were counted to calculate the MLI (=$\frac{\text{pipeline}\;\text{length}}{\text{Ns}}$) with three repetitions.

### 2.12 Immunohistochemistry (IHC)

We retrieved antigens in the samples after dewaxing. These samples were then blocked with 3 % bovine serum albumin buffer and incubated separately with primary antibody [*anti*-IL-1β (#12242, Cell Signaling Technology, MA, USA), *anti*-TNF-α (Cat No. 60291-1-Ig, Proteintech, IL, USA), or anti-cleaved-caspase (-*c*-Casp)-3 (#9661, Cell Signaling Technology, MA, USA)] at 4 °C overnight. Following incubation with horseradish peroxidase-labeled secondary antibody for 50 min, the samples were counterstained with hematoxylin and sealed for imaging under a microscope.

### 2.13 Network pharmacology-based prediction for molecular signaling

Network pharmacology aided in predicting the molecular mechanism of QJHTD action against COPD. The approach involved target fishing, molecular interaction networking, and pathway enrichment analysis, which required the databases, such as TCMSP (https://tcmsp-e.com/), BATMAN-TCM (http://bionet.ncpsb.org.cn/batman-tcm/), PubChem (https://pubchem.ncbi.nlm.nih.gov/), SwissTargetPrediction (http://www.swisstargetprediction.ch/), DisGeNET (http://www.disgenet.org/), CTD (http://ctdbase.org/), GeneCards (https://www.genecards.org/), STRING (version 11.0; https://string-db.org/), DAVID (https://david.ncifcrf.gov/), and KEGG (https://www.genome.jp/kegg/), as well as the software Cytoscape (version 3.7.1; https://cytoscape.org/). The analytical workflow was based on our previous report [18].

### 2.14 RNA sequencing (RNA-seq) and analysis

Lung tissue samples from the mice with or without irritation were sent to the Majorbio Biopharm Technology Co., Ltd. (Shanghai, China) for total RNA extraction, complementary DNA (cDNA) library construction, RNA-seq, quality control of sequencing data, differential gene expression analysis, GO term classification, and KEGG pathway enrichment analysis. The procedure was as reported previously [19]. An adjusted *p* value less than 0.05 denoted significant enrichment. All sequencing data were submitted to the GEO repository (GSE223102).

### 2.15 Nontargeted LC–MS/MS-based metabolomics

Part of the previously collected lung tissue was delivered to Majorbio for nontargeted metabolomic profiling, which included specimen preparation, quality control of sampling, metabolite identification using UPLC-TQ-TOF-MS/MS (AB SCIEX, CA, USA), metabolomics data analysis, and KEGG-based metabolic pathway enrichment. The workflow was based on the previous report [20].

### 2.16 In silico molecular docking

Virtual molecular docking analysis was applied to surmise the therapeutic potency of candidate molecules in QJHTD for COPD targeting. The required databases included RCSB Protein Data Bank (http://www.rcsb.org/) and PubChem. The tool for semiflexible docking was AutoDock (version 4.2.6). The procedure was as reported previously [18].

### 2.17 Statistical analysis

All experimental data were statistically analyzed using Prism (version 8.0; GraphPad, CA, USA) and expressed as the mean ± standard deviation (SD). Statistical significance was assessed using analysis of variance and Tukey‒Kramer post hoc test. In all analyses, a *p* value < 0.05 was considered significant.

## 3 Results

### 3.1 Characteristic fingerprint spectrum of QJHTD

**Fig. 1:**
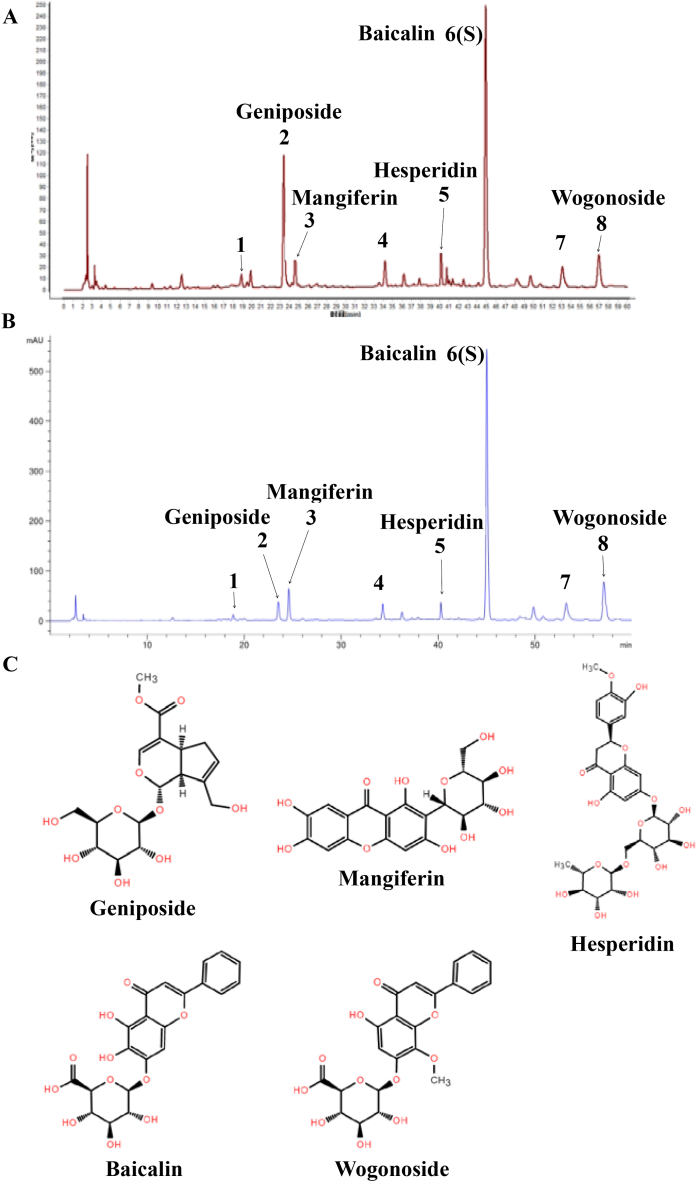
Characteristic fingerprint spectrum of QJHTD. **A** Integrated characteristic fingerprint peaks based on multibatch testing. **B** Characteristic fingerprint peaks of the samples in the present study. Peak 1, an undefined compound; peak 2, geniposide; peak 3, mangiferin; peak 4, an undefined compound; peak 5, hesperidin; peak 6, baicalin (S); peak 7, an undefined compound; peak 8, wogonoside. **C** Molecular structures of the defined compounds in QJHTD.

The typical fingerprints of QJHTD were provided by the Tianjin Institute of Pharmaceutical Research (Tianjin, China). As shown in Fig. 1A, eight shared peaks (peaks 1–8) were designated under various batches of samples, characterizing the chemical profile of QJHTD. Fig. 1B shows the major chemical compositions of the samples in this study. Of these fingerprints, peaks 2, 3, 5, 6, and 8 represented geniposide, mangiferin, hesperidin, baicalin (reference substance, S), and wogonoside, respectively; peaks 1, 4, and 7 were undefined. Molecular structures of the defined compounds in QJHTD are shown in Fig. 1C.

### 3.2 QJHTD exerts cytoprotective and antiinflammatory effects on COPD In vitro

**Fig. 2:**
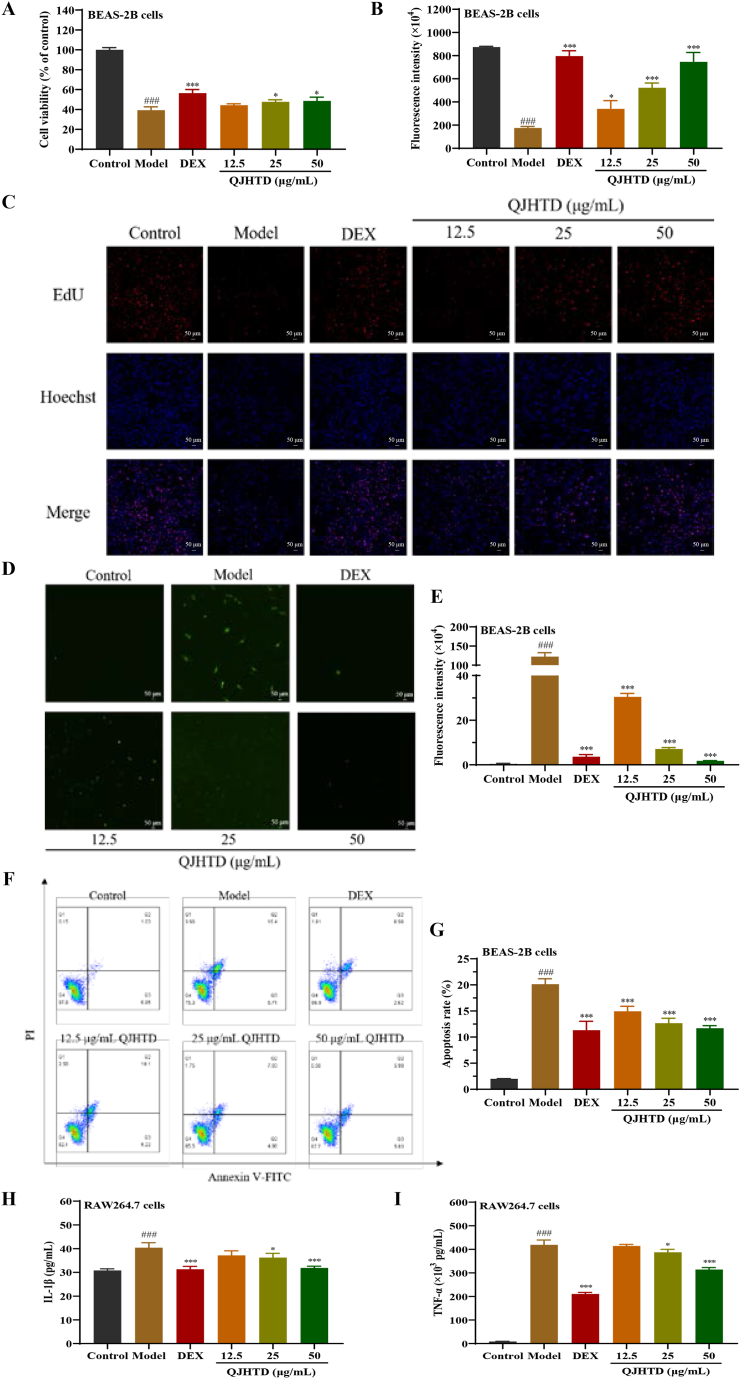
*In vitro* therapeutic effects of QJHTD on COPD. **A** Effect of QJHTD intervention on the viability of CSE–LPS cochallenged BEAS-2B cells. **B** and **C** Effect of QJHTD intervention on the proliferation of CSE–LPS cochallenged BEAS-2B cells. **D**–**G** Effect of QJHTD intervention on the apoptosis of CSE–LPS cochallenged BEAS-2B cells. **H** and **I** Effects of QJHTD intervention on the expression of proinflammatory cytokines (IL-1β and TNF-α) in CSE–LPS cochallenged RAW264.7 cells. All data are expressed as the mean ± SD (*n* = 3). ###*p* < 0.001 *versus* the control group. *p < 0.05, ***p < 0.001 *versus* the model group.

Since airway epithelial cell dysfunction and inflammatory response are the central events in COPD pathophysiology, we surveyed *in vitro* phenotypes including cell viability, proliferation, and apoptosis, as well as inflammatory markers, in the presence or absence of QJHTD intervention to assess the therapeutic potential of QJHTD against COPD. As shown in Fig. 2A, CSE–LPS costimulation significantly inhibited BEAS-2B cell viability, whereas QJHTD (25 and 50 μg mL^−1^) and DEX were conducive to the survival of the damaged BEAS-2B cells. In addition, the EdU assay revealed a significant decrease in the fluorescence intensity of the model compared with that of the control; the injured cells with drug intervention exhibited remarkably higher fluorescence intensities than model cells without treatment (Fig. 2B and (C)), suggesting that QJHTD improved the impaired proliferation of bronchial epithelial cells. The TUNEL assay revealed that the fluorescence intensity in the model group was strikingly higher than that in the control group; QJHTD or DEX involvement markedly attenuated the fluorescent signals from the treated cells as opposed to no drug intervention in the model (Fig. 2D and E). Likewise, the FACS assay showed that irritation significantly elevated the apoptosis rate of BEAS-2B cells, whereas QJHTD or DEX intervention evidently restrained this detrimental alteration (Fig. 2F and G). Taken together, these results demonstrate a potent antiapoptotic property of QJHTD in the CSE–LPS coirritated BEAS-2B cells.

**Fig. 3:**
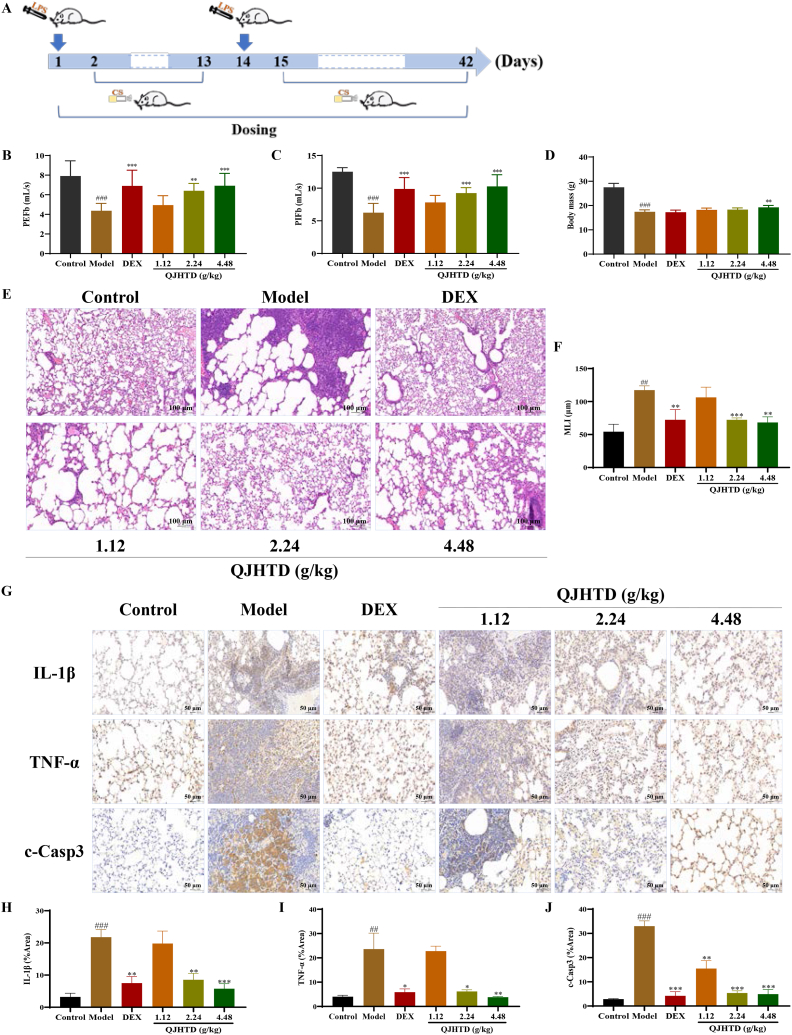
*In vivo* therapeutic effects of QJHTD on COPD. **A** COPD modeling and QJHTD administration in mice. **B** and **C** Effects of QJHTD treatment on the pulmonary function parameters (PEFb and PIFb) of CS–LPS-exposed mice. Data are presented as the mean ± SD (*n* = 8). **D** Effects of QJHTD treatment on the body mass of CS–LPS-exposed mice. Data are presented as the mean ± SD (*n* = 8). **E** and **F** Effect of QJHTD treatment on lung histopathology in CS–LPS-exposed mice. Data are presented as the mean ± SD (*n* = 3). **G**–**J** Effects of QJHTD treatment on the levels of proinflammatory cytokines (IL-1β and TNF-α) and the apoptotic signaling molecule (c-Casp3) in the lung tissue of CS–LPS-exposed mice. Data are presented as the mean ± SD (*n* = 3). ##*p* < 0.01, ###*p* < 0.001 *versus* the control group. *p < 0.05, **p < 0.01, and ***p < 0.001 *versus* the model group.

Regarding inflammatory cell phenotypes, the proinflammatory factors IL-1β and TNF-α are deemed biomarkers for inflammatory activation owing to their sensitivities in COPD progression [21,22]. ELISA revealed that CSE–LPS cochallenge of RAW264.7 cells notably increased IL-1β and TNF-α production in the extracellular medium, reflecting the proinflammatory nature of the CSE–LPS combination. Compared with the model group, the QJHTD (25 and 50 μg mL^−1^)-treated and DEX-treated groups exhibited a substantial reduction in secretory IL-1β and TNF-α levels (Fig. 2H and I), indicating that QJHTD mitigated the respiratory inflammation evoked by CSE and LPS. Collectively, these findings validate the cytoprotective and antiinflammatory effects of QJHTD against COPD.

### 3.3 QJHTD administration improves respiratory function and alleviates lung injury in mice with COPD

COPD modeling and QJHTD administration in mice are as shown in Fig. 3A. PIFb and PEFb values were measured to estimate whether QJHTD improved spontaneous ventilation in COPD mice [23,24]. Final respiratory assessment in this study showed that combined exposure to CS and LPS caused a robust decrease in mouse PIFb and PEFb values, which manifested lung dysfunction following CS–LPS coexposure and was representative of bronchial obstruction in COPD. QJHTD (2.24 or 4.48 g kg^−1^) or DEX administration significantly reversed these downward trends (Fig. 3B and C), indicating that QJHTD relieved airflow limitation in the COPD mice. Given the correlation between COPD severity and histopathology, H&E staining offered morphological evidence supporting QJHTD's effectiveness against COPD. As expected, mice subjected to CS and LPS stimulation showed significantly greater MLI than controls, which suggested that CS–LPS cochallenge impaired bronchioloalveolar walls and contributed to the pathogenesis of emphysema, thereby accounting for reduced PIFb and PEFb values. QJHTD (2.24 and 4.48 g kg^−1^)-treated and DEX-treated groups presented a remarkable decline in MLI compared to the model group (Fig. 3E and F), implying that QJHTD affected the development of COPD on both functional and structural scales. To profile pulmonary inflammatory lesions, we analyzed IL-1β, TNF-α, and c-Casp3 expression levels in mouse lung tissue using IHC. The positive areas labeled by these markers were sharply expanded under exposure to CS and LPS, but prominently shrunk after QJHTD (2.24 or 4.48 g kg^−1^) or DEX treatment (Fig. 3G–J), reconfirming that the therapeutic effects of QJHTD on COPD are relevant to restraint of airway inflammation and bronchoalveolar cell death. However, COPD progression is partly linked to loss of body mass [25]. We thus estimated whether QJHTD administration affected the weight of COPD mice. In the present study, mice undergoing CS and LPS stimulation showed a dramatic drop in body mass. Intriguingly, high-dose QJHTD (4.48 g kg^−1^) prevented body weight loss, whereas DEX produced no significant impact (Fig. 3D). Collectively, the integrative *in vivo* activity against COPD typifies the therapeutic potency of QJHTD, parallel to its performance *in vitro*.

### 3.4 Network pharmacology predicts the molecular mechanism of QJHTD action on COPD is related to modulating inflammatory and apoptotic signaling

**Fig. 4:**
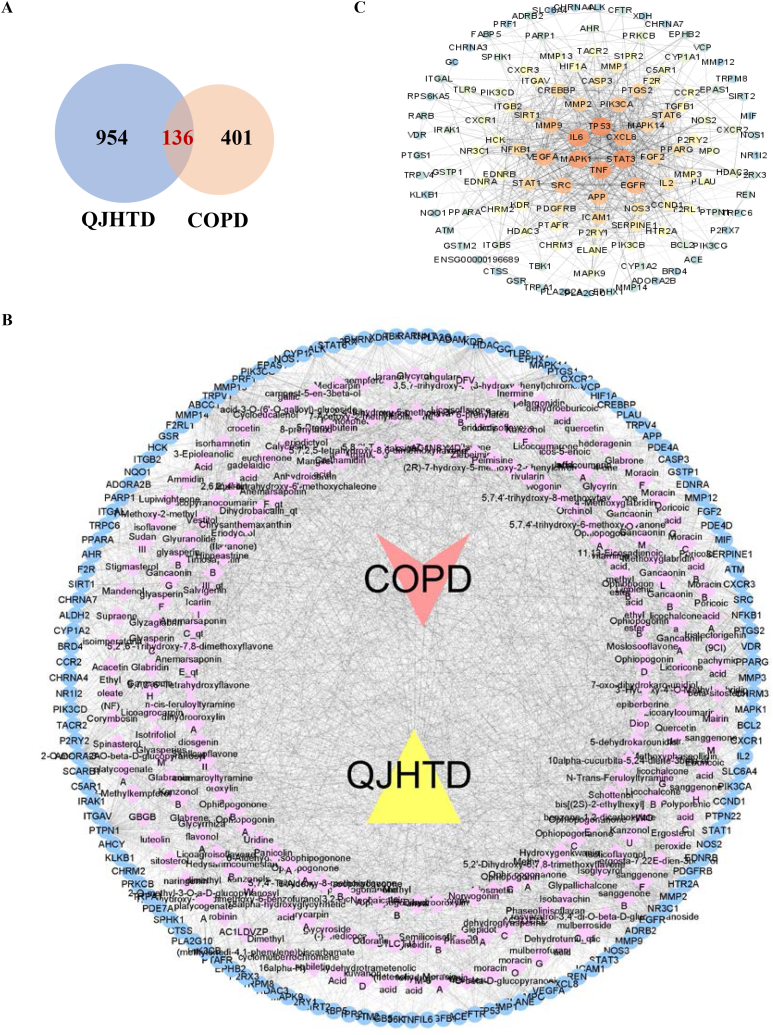
Prediction of constituent–target interplay for the action of QJHTD against COPD. **A** Intersections between QJHTD targets and therapeutic targets for COPD. **B** Drug (QJHTD)–constituent–target–disease (COPD) association network. **C** Protein–protein interaction analysis to identify the hub targets of QJTHD in COPD treatment.

**Fig. 5:**
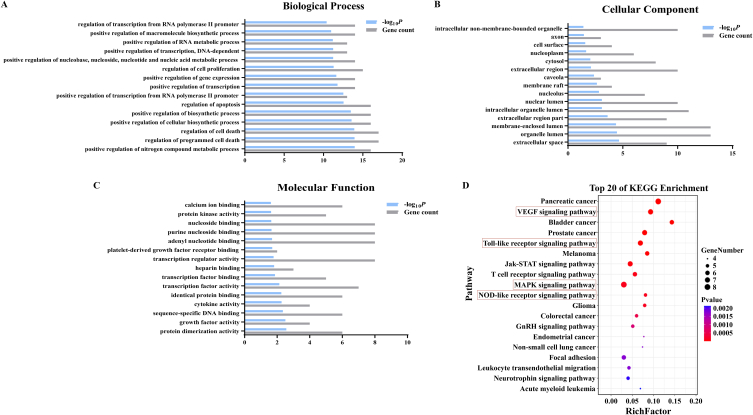
Prediction of GO functions and KEGG pathways related to the action of QJHTD against COPD. **A**–**C** GO terms in biological process (BP), molecular function (MF), and cellular component (CC) domains annotating QJHTD activity against COPD. **D** KEGG pathway enrichment for the potential molecular mechanism of QJHTD activity against COPD.

Next, we tentatively probed the therapeutic machinery conferring QJHTD activity against COPD using network pharmacology. We searched 954 drug targets (from the SwissTargetPrediction database) corresponding to 243 active compounds of QJHTD (from the TCMSP and BATMAN-TCM databases) and 401 therapeutic targets for COPD (integrating 668 therapeutic targets from the database DisGeNET, 19323 from CTD, and 5019 from GeneCards). The Venn diagram (Fig. 4A) exhibited 136 intersections of the two target sets for QJHTD treatment of COPD. These intersections and the active compounds were used to construct a drug–ingredient–target–disease association network consisting of 359 nodes and 3286 edges (Fig. 4B). Moreover, we employed the above intersections to build a PPI network through the STRING database. The PPI network was optimized and topologically analyzed using Cytoscape (Fig. 4C). The results indicated 26 hub targets/proteins, including TP53, STAT3, IL-6, MAPK1, TNF, and CXCL8, under the conditions of degree >18, betweenness >0.00293113, and closeness >0.40878378 (Table S1). The information on the hub proteins was then input into the DAVID database for GO term and KEGG pathway enrichment analyses. In GO term enrichment analysis, the identified hub proteins were linked with 435 terms in biological process (BP) (Fig. 5A), 24 in cellular component (CC) (Figs. 5B), and 29 in molecular function (MF) (Fig. 5C) categories. Of the significantly enriched terms, the representatives in BP were exemplified as follows: regulation of cell death, positive regulation of biosynthetic process, regulation of apoptosis, positive regulation of gene expression, and regulation of cell proliferation (Fig. 5A). KEGG pathway enrichment analysis unveiled latent molecular pathways, including the NOD-like receptor (NLR) signaling pathway, MAPK signaling pathway, Jak-STAT signaling pathway, Toll-like receptor (TLR) signaling pathway, and VEGF signaling pathway (Fig. 5D). These results suggest that the modulation of inflammatory (such as NLR, MAPK, TLR, and VEGF) and cell death-related signaling cascades is involved in the *anti*-COPD action of QJHTD.

### 3.5 Transcriptome analysis identifies DEGs and signaling pathways involved in the Anti-COPD Effect of QJHTD

**Fig. 6:**
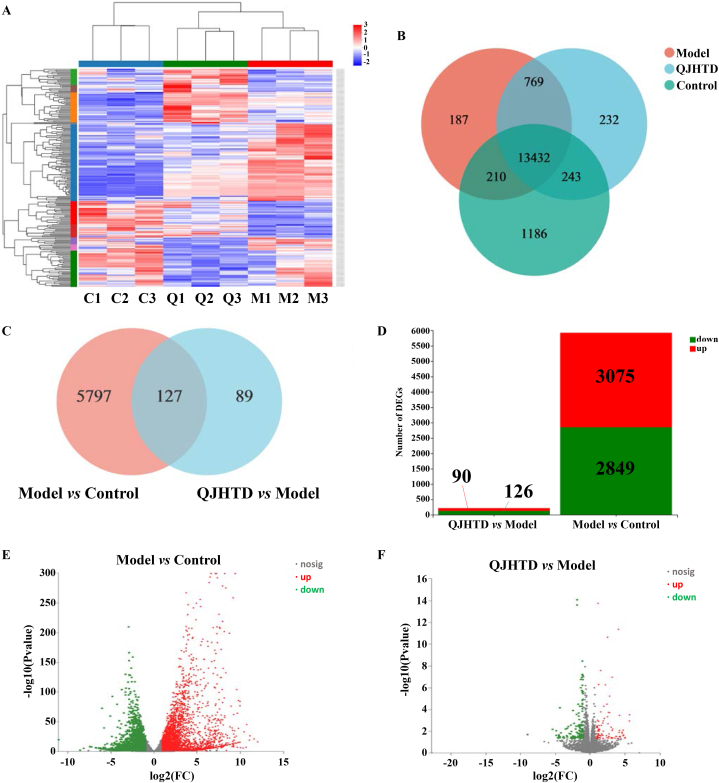
RNA-seq-dependent differential gene expression analysis of the lung tissue of mice with COPD receiving QJHTD regimen. **A** Cluster test of gene expression patterns in the control (C1–C3), model (M1–M3), and QJHTD (Q1–Q3) groups. **B** Expressed gene counts in the control, model, and QJHTD groups. **C** DEG counts in the model-*versus*-control and QJHTD-*versus*-model comparisons. **D**–**F** Analysis of upregulated and downregulated DEGs in the model-*versus*-control and QJHTD-*versus*-model comparisons.

**Fig. 7:**
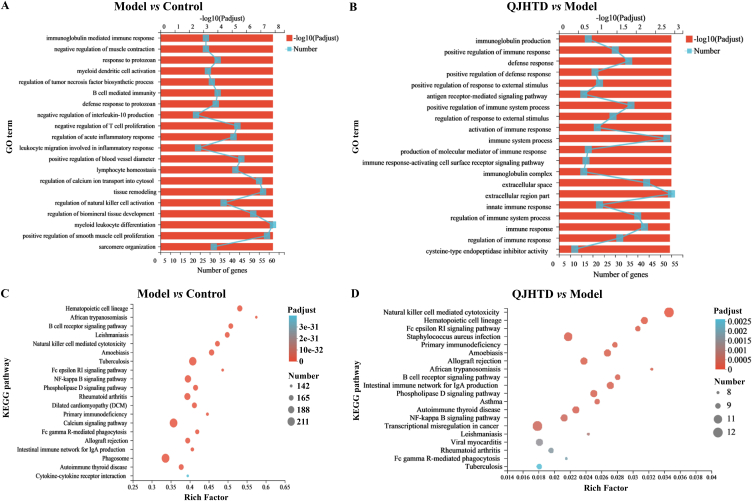
GO function and KEGG pathway enrichment analyses based on the gene sets of intergroup comparisons. **A** and **B** GO terms annotating DEG biofunctions in the model-*versus*-control and QJHTD-*versus*-model comparisons. **C** and **D** KEGG pathways contributing to the modulation of COPD pathogenesis by QJHTD.

Relative to the virtual prediction, RNA-seq provides practical insight into the transcriptome of cells or tissue that decrypts the DEGs in lung tissue of the COPD mice receiving QJHTD treatment (at high/optimal dose, 4.48 g kg^−1^) in the present study. Homogenization of mRNA abundance data showed that TPM values in all samples were not significantly different (Fig. S1), indicating that the mRNA expression data were applicable to differential expression analysis. DEG clustering in the three groups is presented as a heat map in Fig. 6A and as a three-circle Venn diagram in Fig. 6B. DEGs in the model-*versus*-control and QJHTD-*versus*-model comparisons were enumerated using a Venn diagram (Fig. 6C) and stacked bar chart (Fig. 6D) and visualized as volcano plots (Fig. 6E and F), according to the screening criteria of *p* value < 0.05 and |log_2_(FC)| ≥ 1. A total of 5924 DEGs were identified between the model and control groups, involving 3075 upregulated and 2849 downregulated genes. Meanwhile, 216 DEGs were identified between the QJHTD and model groups, involving 90 upregulated and 126 downregulated genes (Fig. 6C and D; Table S2). Subsequently, we implemented GO term and KEGG pathway enrichment analyses to expound DEG functions and deduce the molecular mechanism of action of QJHTD against COPD. Functional annotation in BP showed that the DEGs between the model and control groups were enriched in such terms as regulation of natural killer cell (NKC) activation, leukocyte migration involved in inflammatory response, regulation of acute inflammatory response, and regulation of tumor necrosis factor biosynthetic process (Fig. 7A). These descriptions characterize the active phase or acute exacerbation of chronic inflammation, conforming to the pathophysiology of COPD. The DEGs between the QJHTD and model groups were enriched in the following processes: regulation of immune response, innate immune response, regulation of response to external stimulus, and positive regulation of defense response (Fig. 7B). These results suggest that the efficacy of QJHTD in treating COPD relies on immune adjustment and antiinflammatory interference. The pathway enrichment profile disclosed that the DEGs between the model and control groups participated in a series of molecular signaling cascades, such as the phospholipase D (PLD) signaling pathway, NF-kappa B (NF-κB) signaling pathway, Fc epsilon RI (FcεRI) signaling pathway, and NKC-mediated cytotoxicity (Fig. 7C). Analogously, the DEGs between the QJHTD and model groups contributed to pathways including the NF-κB signaling pathway, PLD signaling pathway, FcεRI signaling pathway, and NKC-mediated cytotoxicity (Fig. 7D). These findings imply that the molecular routes relevant to NF-κB, PLD, and FcεRI, as well as NKC-mediated cytotoxicity (ultimately leading to cell apoptosis), are instrumental in the therapeutic mechanism of QJHTD against COPD.

**Fig. 8:**
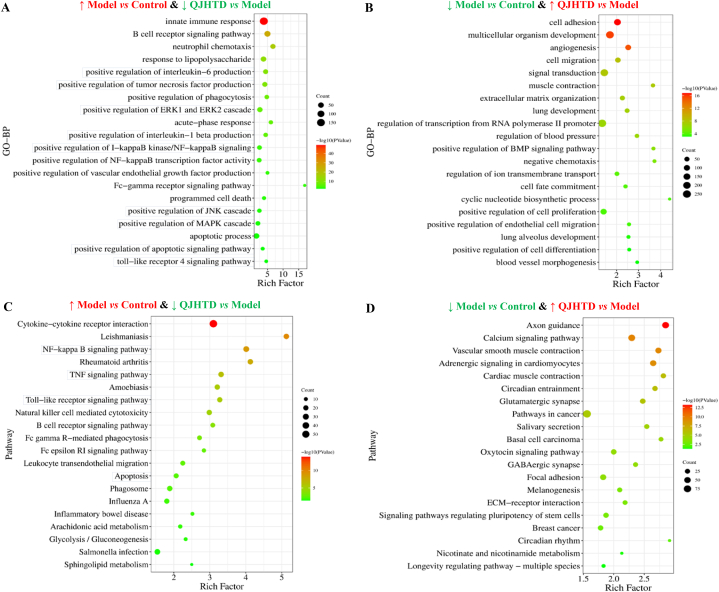
Molecular pathways underlying the *anti*-COPD property of QJHTD. **A** BPs upregulated in the model-*versus*-control and downregulated in the QJHTD-*versus*-model comparison. **B** BPs downregulated in the model-*versus*-control and upregulated in the QJHTD-*versus*-model comparison. **C** Pathways upregulated in the model-*versus*-control and downregulated in the QJHTD-*versus*-model comparison. **D** Pathways downregulated in the model-*versus*-control and upregulated in the QJHTD-*versus*-model comparison.

Furthermore, we investigated whether QJHTD positively or negatively tuned molecular signaling for the remission of COPD. Intersections of the downregulated (upregulated) genes in the model-*versus*-control group comparison and the upregulated (downregulated) genes in the QJHTD-*versus*-model group comparison were used for GO function and KEGG pathway enrichment reanalyses. The functional annotations in BP showed that the processes (or signaling cascades) reinforced in the model group relative to those in the control group and concurrently weakened in the QJHTD group relative to those in the model group embraced positive regulation of IL-6, TNF, and IL-1β production, as well as positive regulation of MAPK (specified as ERK1, ERK2, and JNK), I-kappa B (IκB) kinase/NF-κB, apoptosis, and TLR4 signaling pathways (Fig. 8A). The processes (or signaling cascades) that were inhibited in the model group relative to those in the control group and enhanced in the QJHTD group relative to those in the model group included negative chemotaxis, lung alveolus development, and positive regulation of cell proliferation and differentiation (Fig. 8B). KEGG pathway enrichment analysis showed that the molecular pathways that were upregulated in the model group *versus* the control group and simultaneously downregulated in the QJHTD group *versus* the model group included the NF-κB, TNF, and TLR signaling pathways, and apoptosis (Fig. 8C). The pathways that were downregulated in the model group *versus* in the control group and concurrently upregulated in the QJHTD group *versus* the model group included the calcium signaling pathway, adrenergic signaling in cardiomyocytes, and circadian entrainment (Fig. 8D). Collectively, the landscape of pathway activity regulation suggests that suppressing the molecular routes of IL-1β, IL-6, TNF, IκB–NF-κB, TLR, MAPK (involving ERK1/2 and JNK), and apoptosis is prominent in the *anti*-COPD action of QJHTD, which closely mirrored our speculation based on network pharmacology.

### 3.6 Metabolome profiling Unveils metabolite signature in mice with COPD receiving QJHTD treatment

**Fig. 9:**
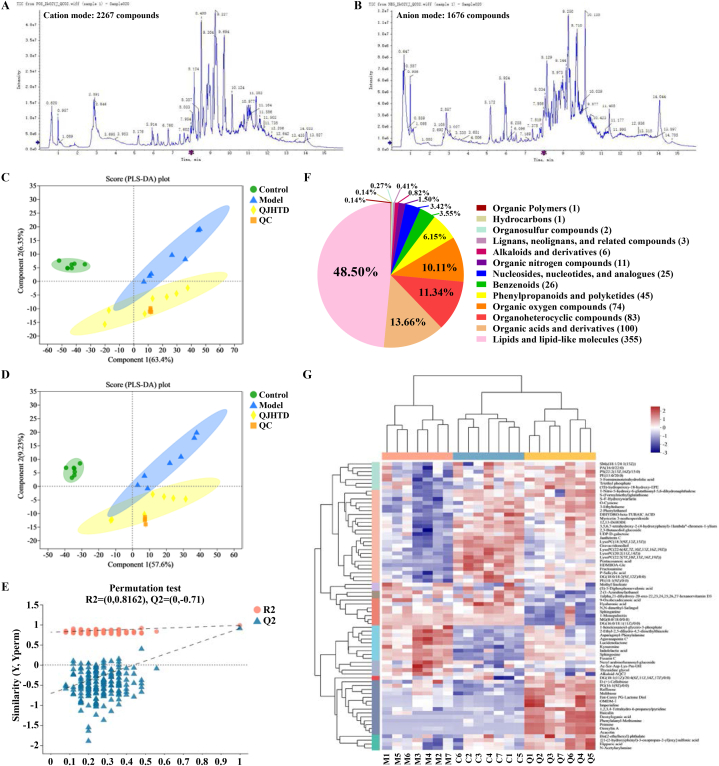
Metabolite outline in metabolic profiling of mouse lung tissue. **A** and **B** Total ion chromatograms (TICs) of quality control samples in the cation and anion modes. **C** and **D** PLS-DA score plots in the cation and anion modes showing the classification of mouse lung tissue samples. **E** Permutation test of the PLS-DA model. **F** HMDB categorization (superclass) of 732 compounds in the metabolomic dataset. Numbers in parentheses represent metabolite counts in the superclass terms. **G** Cluster heatmap visualizing metabolite annotations in the control (C1–C7), model (M1–M7), and QJHTD (Q1–Q7) groups.

**Fig. 10:**
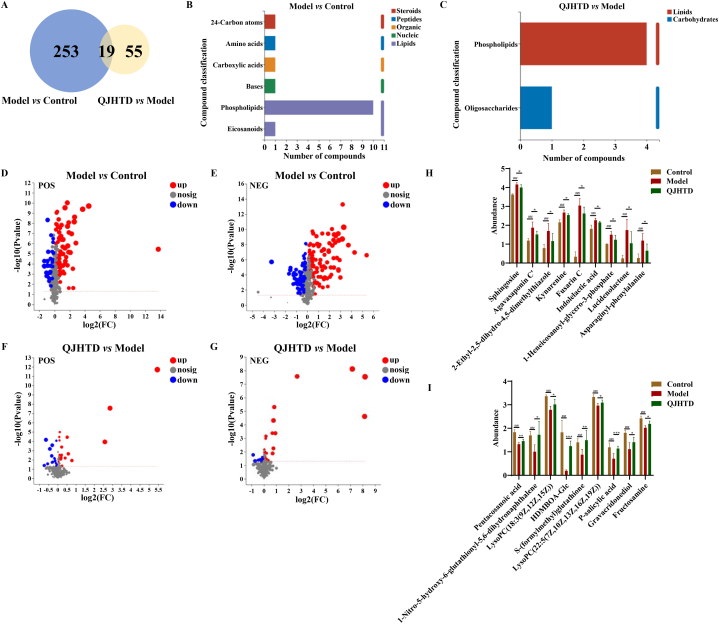
DMB metabolomics analysis of mouse lung tissue. **A** Venn diagram of DMB counts in the model-*versus*-control and QJHTD-*versus*-model comparisons. **B** and **C** KEGG compound classification of DMBs in the model-*versus*-control and QJHTD-*versus*-model comparisons. **D**–**G** Volcano plots in the positive and negative ion modes visualizing DMBs in the model-*versus*-control and QJHTD-*versus*-model comparisons. **H** and **I** Quantitative differences in endogenous metabolites between the control, model, and QJHTD groups. Data are presented as the mean ± SD (*n* = 7). ###*p* < 0.001 *versus* the control group. *p < 0.05, **p < 0.01, ***p < 0.001 *versus* the model group.

Accumulating evidence supports the correlation of metabolic alterations with COPD pathophysiology and treatment [26]. Moreover, traditional herbs or medicinal formulas have been proven effective in redressing dysmetabolism in multiple diseases [27]. Considering this, we attempted to explore the metabolite signature in QJHTD interference (at high/optimal dose, 4.48 g kg^−1^) with COPD at the molecular level. LC‒MS/MS analysis revealed the metabolite composition of mouse lung tissue. Total ion chromatograms of quality control samples in positive and negative ion modes verified the performance of the LC‒MS/MS system, reflecting the expected reliability of measurement (Fig. 9A and B). The permutation test plot showed an appropriate PLS-DA prediction model without overfitting based on the *Y*-intercept of the Q^2^ regression line <0.05 (Fig. 9E). PLS-DA score plots in the positive and negative ion modes showed minor intragroup differences and sharp intergroup distinctions, indicating that COPD modeling remarkably affected metabolites in mouse lung tissue and that QJHTD altered metabolic patterns in the COPD mice (Fig. 9C and D). A total of 732 identified metabolites were subsumed into 13 chemical categories using HMDB (Fig. 9F). The maximal portion of the classification pie was occupied by lipids and lipid-like molecules, totaling 355 compounds and accounting for 48.50 % of all components, indicating the latent dominance of lipids and lipid metabolism in COPD progression and QJHTD action. A cluster heat map was used to visualize the functional relevance and abundance discrepancy of metabolites in the control, model, and QJHTD groups (Fig. 9G). Certain compounds such as imperialine, baicalin, peimine, oroxylin A, and acacetin were highly abundant in the QJHTD group but of low content in the control and model groups. Therefore, these molecules potentially function as active components of QJHTD in COPD treatment. Differential metabolite (DMB) analysis (Fig. 10A; Tables S3 and S4) revealed 272 DMBs (101 in the positive and 171 in the negative ion mode) between the model and control groups, and 74 DMBs (36 in the positive and 38 in the negative ion mode) between the QJHTD and model groups. These DMBs were chemically classified using the KEGG compound database. Six chemical categories were detected in the model-*versus*-control group comparison and two were detected in the QJHTD-*versus*-model group comparison (Fig. 10B and C). The category phospholipids was shared in the two comparisons, which highlighted the regulatory roles of lipids, specifically phospholipids, in the *anti*-COPD action of QJHTD. We further analyzed the up- and downregulated DMBs in QJHTD treatment of COPD, according to the filter criterion of fold change (FC) > 1, signifying upregulation of the DMBs in the model (QJHTD)-*versus*-control (model) comparison, or FC < 1, denoting the contrary modulation. The results were visualized as volcano plots, exhibiting 178 upregulated and 94 downregulated DMBs in the model-*versus*-control comparison, as well as 50 upregulated and 24 downregulated DMBs in the QJHTD-*versus*-model comparison (Fig. 10D–G). Nine upregulated DMBs in the model-*versus*-control comparison and downregulated DMBs in the QJHTD-*versus*-model comparison overlapped, including sphingosine (Sph), agavasaponin C’, 2-ethyl-2,5-dihydro-4,5-dimethylthiazole, kynurenine, fusarin C, indolelactic acid, 1-heneicosanoyl-*glycero*-3-phosphate, lucidenolactone, and asparaginyl-phenylalanine (Fig. 10H). In addition, nine downregulated DMBs in the model-*versus*-control comparison and upregulated DMBs in the QJHTD-*versus*-model comparison overlapped, encompassing pentacosanoic acid, 1-nitro-5-hydroxy-6-glutathionyl-5,6-dihydronaphthalene, lysoPC(18:3 (9Z,12Z,15Z)), HDMBOA-Glc, *S*-(formylmethyl)glutathione, lysoPC(22:5 (7Z,10Z,13Z,16Z,19Z)), *p*-salicylic acid, gravacridonediol, and fructosamine (Fig. 10I). Together, these endogenic compounds formed a metabolic bridge between COPD development and QJHTD action.

**Fig. 11:**
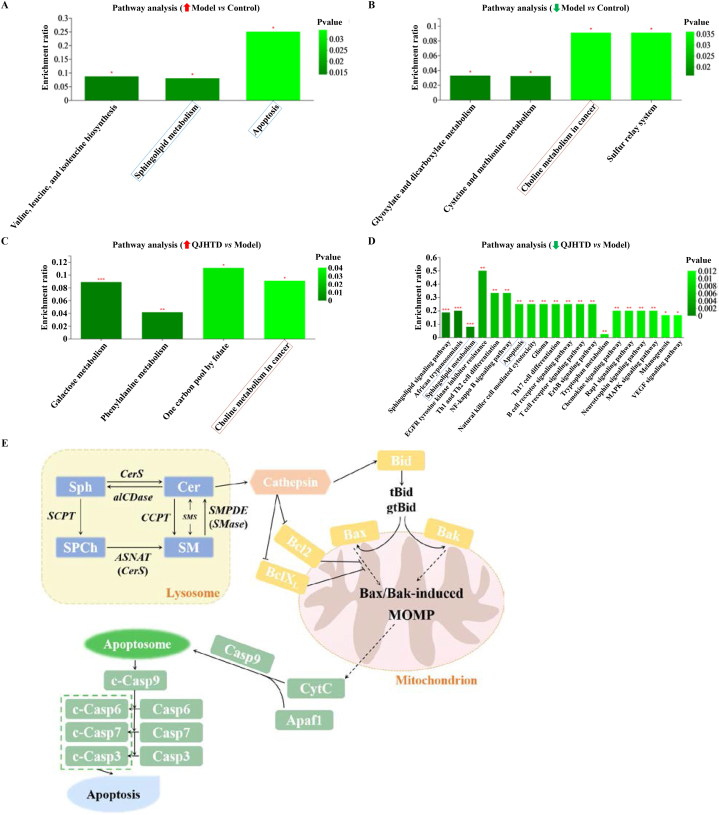
Pathway enrichment analysis of DMBs in intergroup comparisons. **A** and **D** Metabolic pathways upregulated in the model-*versus*-control and downregulated in the QJHTD-*versus*-model comparison. **B** and **C** Metabolic pathways downregulated in the model-*versus*-control and upregulated in the QJHTD-*versus*-model comparison. **E** Nexus between sphingolipid metabolism and apoptosis. *p < 0.05, **p < 0.01, and ***p < 0.001.

Furthermore, KEGG enrichment analysis of up- (down-)regulated DMBs in the model-*versus*-control comparison and concurrently down- (up-)regulated DMBs in the QJHTD-*versus*-model comparison was implemented to identify metabolism-related pathways that mediated the action of QJHTD on COPD. The activities of two pathways, namely, apoptosis and sphingolipid (SL) metabolism, were reinforced in the model-*versus*-control comparison but suppressed in the QJHTD-*versus*-model comparison (Fig. 11A and D). Activity of the pathway choline (Ch) metabolism in cancer was restrained in the model-*versus*-control comparison and concurrently boosted in the QJHTD-*versus*-model comparison (Fig. 11B and C). These findings imply that suppression of apoptosis and SL metabolism with promotion of Ch metabolism partly supports the *anti*-COPD properties of QJHTD, rehighlighting the regulatory significance of SL metabolism.

### 3.7 Integrated Transcriptome–Metabolome analysis reveals the role of SL metabolism in QJHTD action against apoptosis

Comprehensive analysis of metabolomic and transcriptomic data illustrated the link between transcriptional and metabolic controls by QJHTD. The apoptosis pathway was emphasized in that it was enriched with the differential metabolite Sph and emerged at the nexus of the signaling cascades and metabolic pathways (Fig. 11A and D). Using the KEGG pathway IDs of apoptosis (map 04210) and Sph-associated cascades (map 00600, map 01100, and map 04071), we parsed the KEGG pathway maps and edited the regulative network of Sph-originated apoptosis signaling by QJHTD. As shown in Fig. 11E, Sph-induced apoptosis relies on the direct or indirect transmutation of Sph into ceramide (Cer) in lysosomes. Precisely, Sph is converted into Cer through ceramide synthetase (CerS) catalysis or transformed into sphingosyl-phosphocholine (SPCh), sphingomyelin (SM), and Cer in turn under successive catalysis by sphingosine cholinephosphotransferase (SCPT), acyl-CoA:sphingosine *N*-acyltransferase (ASNAT, equivalent of CerS), and sphingomyelin phosphodiesterase (SMPDE, equivalent of sphingomyelinase, SMase). Furthermore, alkaline ceramidase (alCDase) catalyzes the transformation of Cer to Sph. Ceramide cholinephosphotransferase (CCPT) activation enables Cer to convert to SM, while sphingomyelin synthase (SMS) catalyzes the bidirectional conversion between Cer and SM. Lysosomal Cer activates the aspartic protease cathepsin, thereby provoking Bax/Bak-mediated mitochondrial outer membrane permeabilization (MOMP) by upregulating the Bid–Bax/Bak signaling axis and inhibiting Bcl2/BclX_L_ activity. MOMP drives the release of cytochrome-C (CytC) which participates in apoptosome formation by complexing Apaf 1 and pro-Casp9. Active Casp9 cleaves downstream pro-caspases such as pro-Casp 7, pro-Casp3, and pro-Casp 6 to execute cell death. In this diagram, targeting Sph transmutation-relevant enzymes is expected to be part of QJHTD action against bronchoalveolar cell apoptosis in COPD owing to the apparent discrepancies in Sph content and apoptotic factor expression between the COPD mice treated with and without QJHTD.

### 3.8 Discovery of exogenous compounds in lung tissue Ascertains the potential of active ingredients of QJHTD for COPD treatment

**Fig. 12:**
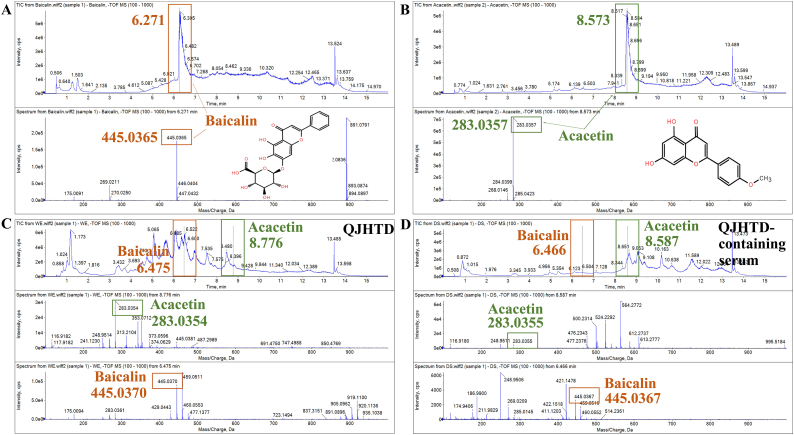
**Potential active components in QJHTD.** **A** and **B** Retention time and mass-to-charge ratio (*m*/*z*) of baicalin and acacetin in mass spectrum analysis. **C** and **D** Mass spectrum analyses of QJHTD preparation and QJHTD-containing serum.

**Fig. 13:**
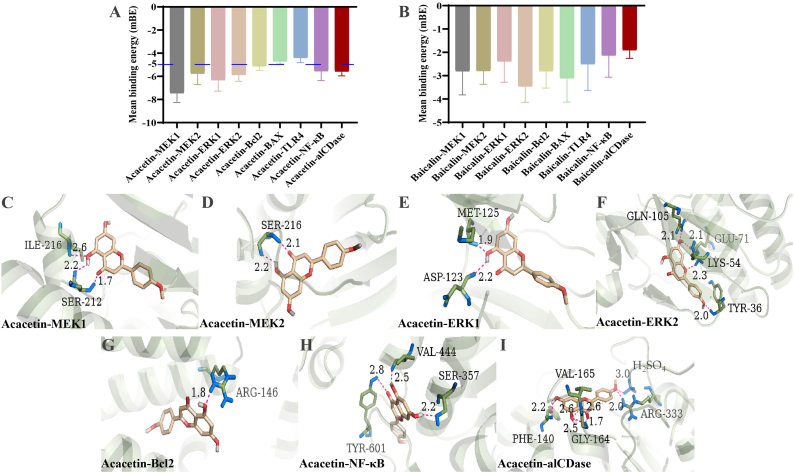
**Interactions of potential active components with target proteins**. **A** and **B** Mean binding energy (mBE) of baicalin and acacetin to targets relevant to inflammation, apoptosis, and sphingolipid metabolism. **C–I** Visualization of acacetin binding to the detected targets with mBE values below −5.

Following the metabolic landscape, extrinsic compounds in the lung tissue of the QJHTD-treated mice, including acacetin, deoxyloganic acid, peimine, oroxylin A, and baicalin, were uncovered. To trace acacetin and baicalin as representative components in the QJHTD preparation, we qualitatively examined these two compounds in QJHTD drug-containing serum and QJHTD samples using LC‒MS (Shimadzu, Japan). Both acacetin and baicalin were detected in the drug-containing serum and the preparation, indicating that these compounds are likely associated with the *anti*-COPD properties of QJHTD *in vitro* and *in vivo* (Fig. 12A–D). Virtual molecular docking allows for inferring the binding of small molecules with target proteins. Thus, we used it to predict the therapeutic potential of acacetin and baicalin against COPD. Herein, we docked acacetin and baicalin on the signaling proteins MEK1, MEK2, ERK1, ERK2, Bcl2, Bax, TLR4, and NF-κB, as well as the metabolic enzyme alCDase. The mean binding energy (mBE) of 10 conformations in one docking study denoted the overall affinity between the ligand and target. The mBE values in acacetin binding to MEK1, MEK2, ERK1, ERK2, Bcl2, NF-κB, and alCDase were less than −5 (absolute values > 5), signifying that acacetin may bind those targets with high affinity (Fig. 13A). The mBE values for acacetin binding to Bax and TLR4 exceeded −5 (absolute values < 5), implying low-affinity interactions between acacetin and these two proteins. In contrast, baicalin showed low binding affinity to all these proteins due to mBE values exceeding −5 (absolute values < 5), suggesting that baicalin may not target those proteins (Fig. 13B). Structurally, the high-affinity interactions of acacetin with the seven targets were visualized, and the docking parameters are presented (Fig. 13C–I; Table 4). These docking parameters portrayed the optimal interaction patterns apt to excellent binding energies. We thus suppose that acacetin harnesses MEK1/2–ERK1/2 signaling, Bcl2 function, NF-κB signaling, and alCDase activity. In other words, acacetin, as an active component, contributes to the antiinflammatory, antiapoptotic, and Sph-anabolism-suppressive effects of QJHTD. Baicalin may exert a curative impact on COPD via alternative routes.

**Table 4:** Docking parameters in the high-affinity interactions of acacetin with seven targets.

Target protein	Bond-forming residue	Hydrogen-bond length	Mean binding energy (mBE)
MEK1	ILE-216	2.6 Å	−7.44
	SER-212	2.2 Å; 1.7 Å	
MEK2	SER-216	2.1 Å; 2.2 Å	−5.74
ERK1	MET-125	1.9 Å	−6.31
	ASP-123	2.2 Å	
ERK2	GLN-105	2.1 Å	−5.87
	GLU-71	2.1 Å	
	LYS-54	2.3 Å	
	TYR-36	2.0 Å	
Bcl2	ARG-146	1.8 Å	−5.11
NF-κB	VAL-444	2.5 Å	−5.54
	TYR-601	2.8 Å	
	SER-357	2.2 Å	
alCDase	PHE-140	2.2 Å	−5.57
	VAL-165	2.6 Å; 2.6 Å	
	GLY-164	2.5 Å; 1.7 Å	
	ARG-333	2.0 Å	
	Sulfuric acid (H_2_SO_4_)	3.0 Å	

## 4 Discussion

COPD is a complex disease that implicates multiple pathologies at the organ and cell levels. Hierarchically, cellular injuries disrupt airway homeostasis, inducing structural abnormalities and obstructive ventilatory defects [28]. In the present study, the combined use of *in vitro*, *in vivo*, and *in silico* assays permitted gathering multidimensional evidence for COPD-like phenotypes and the efficacy of QJHTD regimen against them. BEAS-2B cells and RAW264.7 cells irritated by CSE and LPS were regarded as rational to mimic airway epithelial cell death and cellular inflammatory response, respectively [29]. Male BALB/c mice exposed to CS and LPS were used to imitate COPD development and lung pathology [30]. The *in vitro* and *in vivo* phenotypes from COPD treatment with QJHTD provided cues for mechanistic illustration using network pharmacology, transcriptomics, and metabolomics. Active compounds from the QJHTD extract were identified via comparative LC‒MS analysis of the preparation, drug-containing serum, and lung tissue. The latent activities of these compounds were speculated using molecular docking. The results of all these analyses offered insight into lung distribution-based drug discovery from traditional medicines, accurately deciphering their pharmacodynamic material bases against COPD.

Of note, airway inflammation that is marked by proinflammatory cytokines intensifies with COPD progression, affecting other manifestations of the disease [31]. Apoptosis of airway epithelial cells is deemed equally imperative in COPD pathogenesis, owing to its close correlation with inflammatory outcomes and aggravation rather than simply being a result of CS–LPS irritation [32]. Hence, it is essential to comprehensively understand QJHTD efficacy against COPD in view of its complex pathology. Here, we discovered the combined occurrence of airway inflammation and epithelial cell apoptosis, as evidenced by enhanced cytokine expression and apoptotic phenotypes *in vitro* and *in vivo*. Moreover, CSE–LPS challenge of BEAS-2B cells repressed their viability/proliferation, hinting at the link between lung dysfunction and airway epithelial repair deficiency. The resultant lower values of PIFb and PEFb in the COPD mice *versus* the control group were based on integration of these CS/CSE–LPS-induced lesions, consistent with the histopathological status of enlarged alveoli (increased MLI), decreased alveolar septa, and extensive inflammatory cell infiltration plus exudation. Similar to previous reports on the study of TCM preparations against this disease [33,34], our findings verified that QJHTD exerted beneficial effects on respiratory function and lung histology in subjects with COPD because of its antiinflammatory, antiapoptotic, and cell viability-/proliferation-promoting activities. This deduction opened a new path to explore the putative mechanism of action of QJHTD against COPD, that is, inhibition of inflammatory and apoptotic signaling pathways.

Classical inflammatory signaling involves ILs, TNFs, TLRs, NF-κB, and MAPK activities, and apoptotic signaling involves Bax, Bcl2, Casp9, and Casp3 expression. Crosstalk between the two pathways underlies the inflammation–apoptosis interplay and candidate targets for QJHTD treatment. Targeting these signaling molecules promises to remit airway inflammation and obstruction. A network pharmacology-based strategy predicted that the pathways related to NLR, MAPK, TLR, VEGF, and cell death mediated the therapeutic action of QJHTD on COPD. Transcriptome analysis using RNA-seq revealed that molecular signals such as IL-1β, IL-6, TNF, IκB–NF-κB, TLR, MAPK (involving ERK1/2 and JNK), and apoptosis were intensified in COPD mice compared with controls but weakened in the QJHTD group *versus* the model group. Together, these results underscore the synergistic modulation of proinflammatory cytokines, MAPK signaling, TLR4–NF-κB cascade, and apoptotic factors by QJHTD.

Endogenous metabolism, another aspect of pathogenesis, severity biomarkers, and feasible medication guidance in chronic lung inflammation, has garnered much attention [35,36]. QJHTD-mediated metabolic regulation of COPD lung tissue embodied the restraint on apoptosis and SL metabolism, as opposed to the boost of Ch metabolism. In patients with COPD, enhanced production of Sph as a central metabolic intermediate of SLs/SM activates Casp3 and facilitates PKCδ KD release, leading to cell apoptosis [37,38]. Likewise, Sph elevation elicits IL-1β secretion dependent on the NLRP3 inflammasome from macrophages [39]. Therefore, we deduce that QJHTD can inhibit the switch from SLs/SM to Sph and consequently reduce the levels of metabolite-derived apoptotic/inflammatory mediators on the basis of our findings that lung Sph level was raised due to CS–LPS challenge but lowered after QJHTD treatment. Furthermore, Ch is instrumental in COPD development, especially in bronchospasm for acetylcholine (ACh) synthesis and release [40]. Dampening cholinergic activity is a potent strategy for relieving airway obstruction. In this study, the levels of two Ch metabolites, lysoPC(18:3 (9Z,12Z,15Z)) and lysoPC(22:5 (7Z,10Z,13Z,16Z,19Z)), were decreased in COPD lung tissue but increased post QJHTD administration. We infer that QJHTD intervention may transform Ch into lyso-phosphatidylcholines (lyso-PtdChs) and indirectly block Ch conversion to ACh, mitigating bronchospasm in COPD.

Integrated transcriptomic–metabolomic analysis uncovered the antiapoptotic mechanism of QJHTD compounds partly by targeting Sph-metabolizing enzymes, as QJHTD intervention affected three functional modules, namely, Sph transmutation/cycle, MOMP, and apoptosis-executing cascade, to suppress cell death. This result underlines the implication of SL metabolism in apoptosis signaling and denotes the Sph conversion-targeting antiapoptotic property of QJHTD. Nevertheless, these results fail to explain the antiinflammatory activity of QJHTD pertinent to acting on endogenous metabolism, implying that the antiinflammatory effect of this formula may be independent of metabolic control.

Intriguingly, the representative components acacetin and baicalin from certain medicinal plants were identified in the lung tissue of QJHTD-treated mice. Besides, these two compounds were discerned in the serum from the same mice as well as in the QJHTD preparation used for cellular tests. Thus, acacetin and baicalin may function as active ingredients favoring the *in vitro* and *in vivo* therapeutic efficacy of QJHTD against COPD [41]. Mechanistically, acacetin could bind MEK1/2, ERK1/2, Bcl2, NF-κB, and alCDase according to the results of our docking analysis, which elucidates the signaling modulation by QJHTD and indirectly testifies to the role of acacetin as an effective compound in this preparation. However, molecular docking exhibited low affinity between baicalin and target proteins, indicating that baicalin may exert its action on COPD via distinct signaling cascades, as previously reported [[42], [43], [44]]. Overall, acacetin and baicalin may act synergistically via complementary molecular routes to assist in the *anti*-COPD properties of QJHTD.

## 5 Conclusion

In summary, the present investigation confirmed that QJHTD involvement suppressed airway epithelial cell apoptosis and inflammatory cell activation both *in vitro* and *in vivo*, which was central to COPD remission and connoted the putative action mechanisms upon the apoptotic and inflammatory cascades. Network pharmacology-based prediction and transcriptome analysis emphasized IL-1β, IL-6, TNF, IκB–NF-κB, TLR, MAPK, and apoptosis, detailing QJHTD-triggered molecular signaling. Metabolome profiling, integrated transcriptomic-metabolomic analysis, and virtual molecular docking uncovered the promising active compounds such as baicalin and acacetin in the preparation, as well as the functional cohesion between QJHTD-targeted SL metabolism and apoptosis pathway. These findings provide comprehensive insight into the mechanism of action of QJHTD, highlighting its potential as an alternative to current treatment guidelines.

## Ethics statement

For comprehensive information on ethical guidelines for journal publication, we can refer to the Publishing & Research Ethics section and the Ethics Model on the Researcher Academy. Animal experiments in this study were authorized by the Center for Experimental Animal of Southwest Medical University, and all experimental procedures were approved by the Ethics Committee of Southwest Medical University (license No. 20180309091).

## Funding

This study was funded by the Sichuan Hospital Association Research Fund for Young Pharmacists (Grant No. 22007), the 10.13039/501100001809National Natural Science Foundation of China (Grant No. 81804221), the National Major Science and Technology Project of the Ministry of Science and Technology of China (Grant No. 2018ZX09721004-006), and the Sichuan Province Science and Technology Program (Grant No. 2019YJ0473).

## Institutional review board statement

This study was conducted in accordance with ARRIVE guidelines and approved by Ethics Committee of Southwest Medical University (license No. 20180309091).

## Informed consent statement

Not applicable.

## Data availability statement

Data to support the study are contained within the article and Supplementary Materials. Data associated with this study has been deposited into a publicly available repository GEO repository (GSE223102).

## CRediT authorship contribution statement

**Jing Yang:** Writing – original draft, Funding acquisition, Conceptualization. **Xin Shen:** Methodology, Investigation. **Mi Qin:** Methodology, Investigation. **Ping Zhou:** Methodology, Investigation. **Fei-Hong Huang:** Methodology, Investigation. **Yun You:** Supervision. **Long Wang:** Formal analysis, Data curation. **Jian-Ming Wu:** Supervision, Resources, Funding acquisition.

## Declaration of competing interest

The authors declare that they have no known competing financial interests or personal relationships that could have appeared to influence the work reported in this paper.

## Figures and Tables

**Fig. S1:**